# Supplementary material for: Workplace discrimination as risk factor for long-term sickness absence: Longitudinal analyses of onset and changes in workplace adversity
Source: PLoS One. 2021 Aug 5;16(8):e0255697. doi: 10.1371/journal.pone.0255697 (PMC8341535; doi:10.1371/journal.pone.0255697)
Supplement: S1 Fig — N; number of employees, n; number of employee-observations, LTSA; long-term sickness absence. (PDF) [file pone.0255697.s001.pdf]

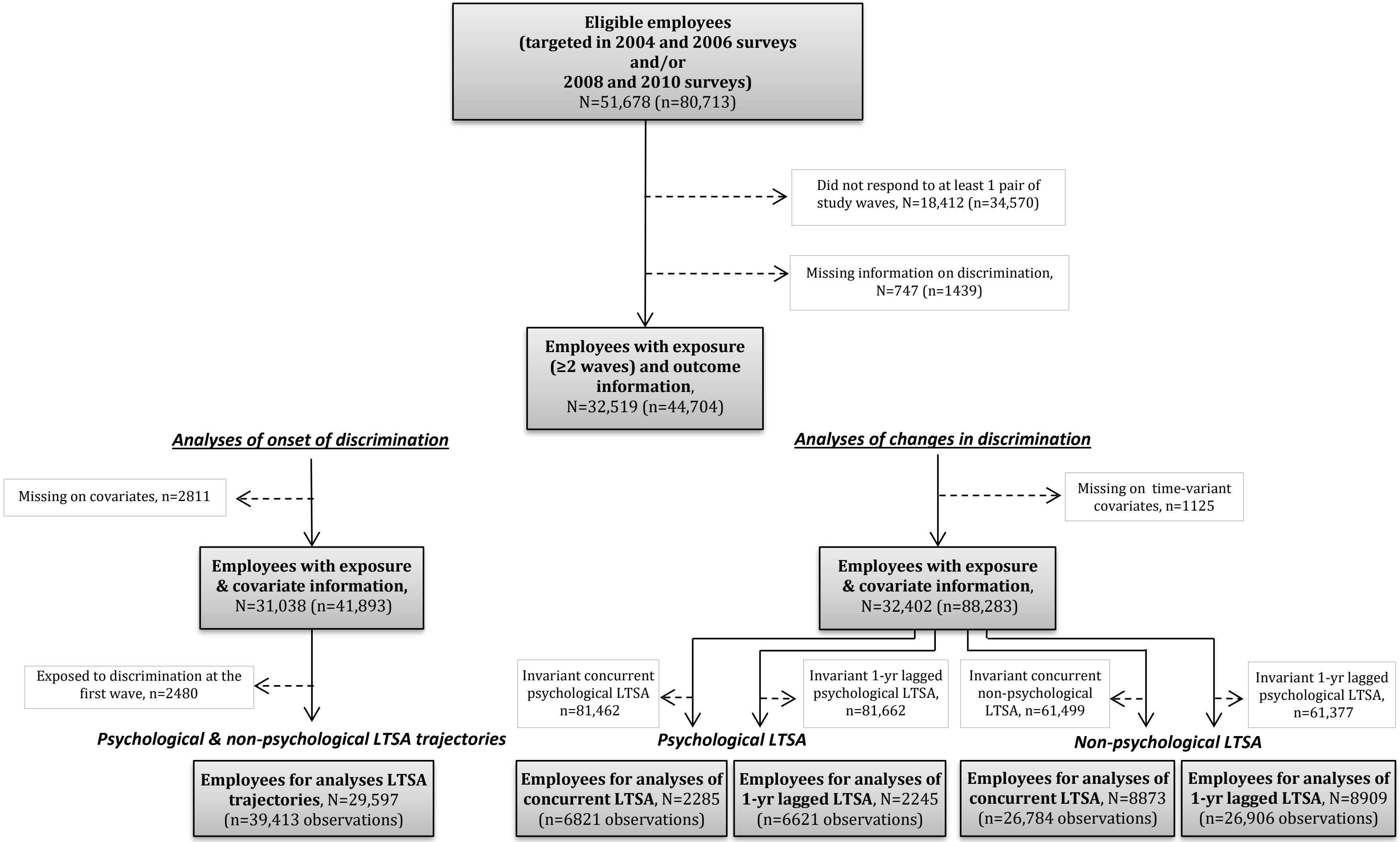

S1 Fig. Flowchart showing the selection of eligible FPS employees for the analyses of onset of and changes in workplace discrimination and long-term sickness absence. N; number of employees, n; number of employee-observations, LTSA; long-term sickness absence.
