# Supplementary material for: Workplace discrimination as risk factor for long-term sickness absence: Longitudinal analyses of onset and changes in workplace adversity
Source: PLoS One. 2021 Aug 5;16(8):e0255697. doi: 10.1371/journal.pone.0255697 (PMC8341535; doi:10.1371/journal.pone.0255697)
Supplement: S2 Fig — (DOCX) [file pone.0255697.s002.docx]

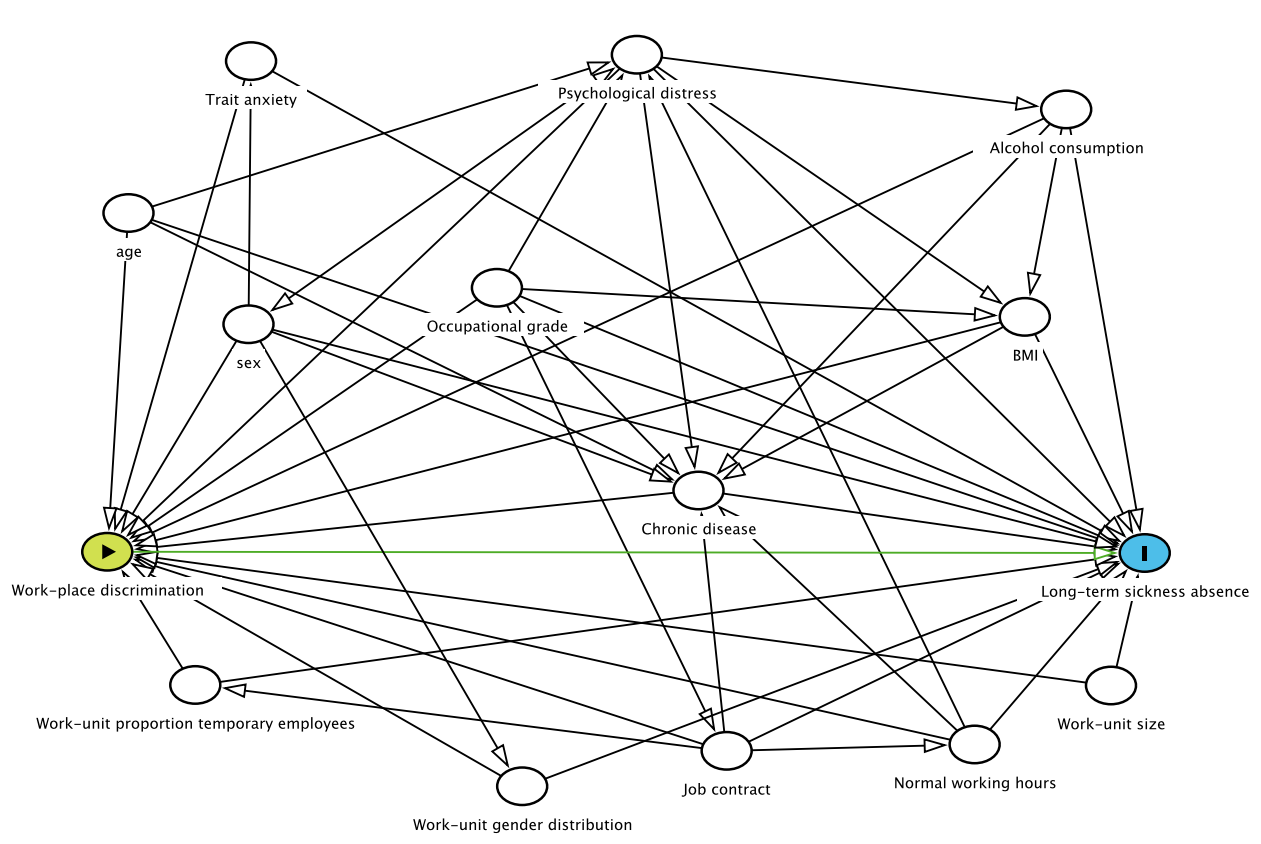


**S2 Figure.** Directed acyclic graph of the assumed causal network between work-place discrimination and long-term sickness absence
