# Supplementary material for: Workplace discrimination as risk factor for long-term sickness absence: Longitudinal analyses of onset and changes in workplace adversity
Source: PLoS One. 2021 Aug 5;16(8):e0255697. doi: 10.1371/journal.pone.0255697 (PMC8341535; doi:10.1371/journal.pone.0255697)
Supplement: S3 Fig — Prevalence of long-term sickness absence among employees with and without onset of workplace discrimination as well as the risk ratio (RR) and 95% CI’s associated with onset of workplace discrimination. (DOCX) [file pone.0255697.s003.docx]

S3 Figure. Sensitivity analysis by using imputed data on missing covariates. Prevalence of long-term sickness absence among employees with and without onset of workplace discrimination as well as the risk ratio (RR) and 95% CI’s associated with onset of workplace discrimination.


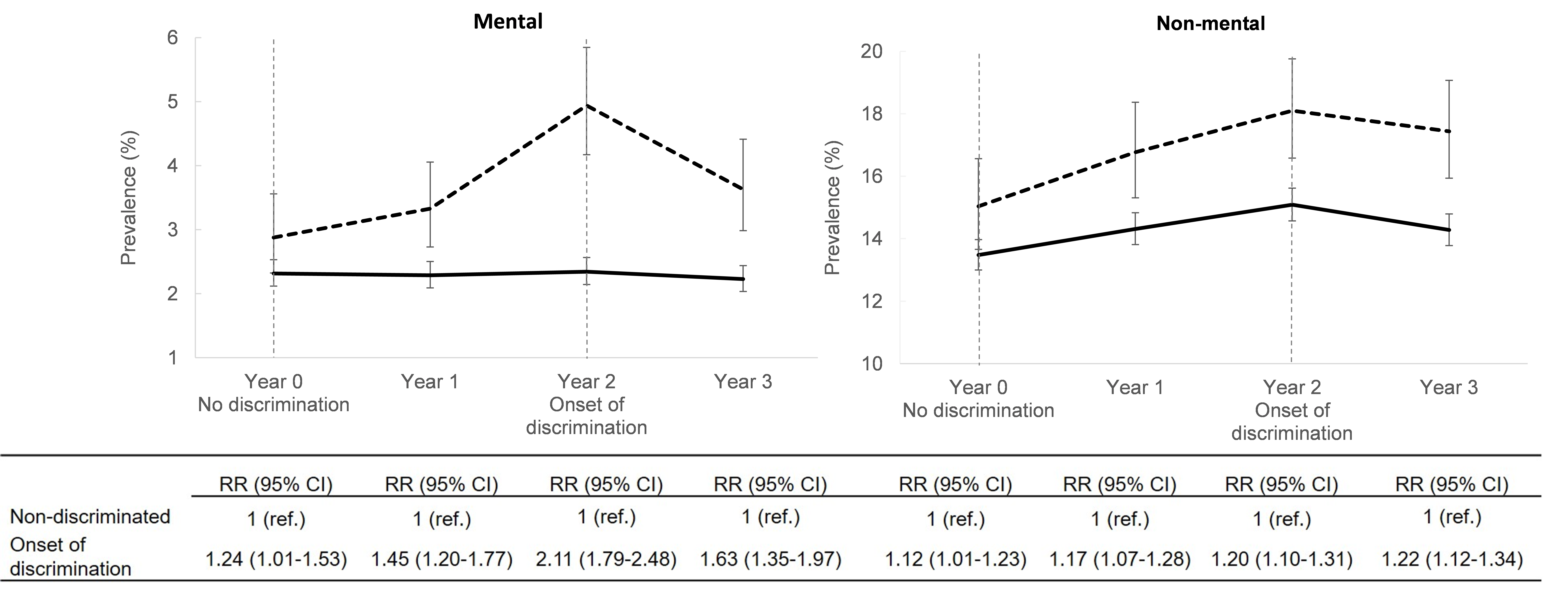


Log-binomial regression analyses adjusted for the following variables at year 0: age, sex, chronic disease, psychological distress, anxiety, BMI, alcohol consumption, shift work, employment contract, occupational grade, work-unit size, work-unit temporary employment, work-unit gender distribution, as well as data-cycle number. Solid lines represents those with onset of workplace discrimination and dashed lines represents those who were not discriminated.
