# Supplementary material for: Workplace discrimination as risk factor for long-term sickness absence: Longitudinal analyses of onset and changes in workplace adversity
Source: PLoS One. 2021 Aug 5;16(8):e0255697. doi: 10.1371/journal.pone.0255697 (PMC8341535; doi:10.1371/journal.pone.0255697)
Supplement: S1 Table — (PDF) [file pone.0255697.s004.pdf]

S1 Table. Measurement time-points for the analyses of onset of and changes in workplace discrimination from the Finnish Public Sector study.

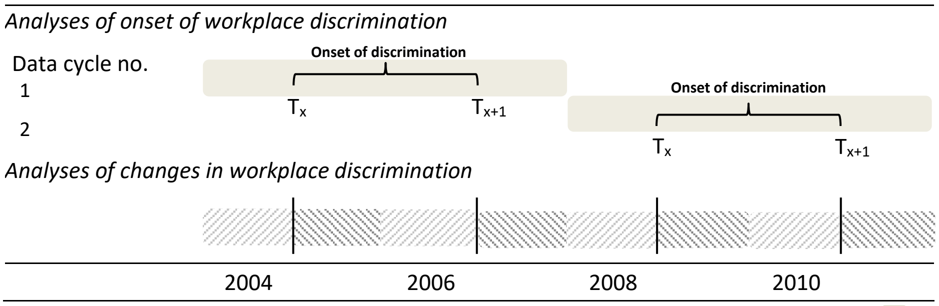

T_x; Employees not exposed to discrimination. T_{x+1}; Onset of discrimination was was measured. Assesment of yearly sickness absence prevalence. Concurrent case or control time. 1-yr lagged case or control time. Assesment of discrimination within the previous year.
