# Supplementary material for: Workplace discrimination as risk factor for long-term sickness absence: Longitudinal analyses of onset and changes in workplace adversity
Source: PLoS One. 2021 Aug 5;16(8):e0255697. doi: 10.1371/journal.pone.0255697 (PMC8341535; doi:10.1371/journal.pone.0255697)
Supplement: S2 Table — Prior long-term sickness absence and odds for onset of discrimination. (DOCX) [file pone.0255697.s005.docx]

**S2 Table. Supplementary analysis. Prior long-term sickness absence and odds for onset of discrimination.**

|  | **Onset of discrimination** | |
| --- | --- | --- |
|  | Cases/No. of observations | OR (95% CI) |
| **Mental diagnosed long-term sickness absence 1-year prior to baseline** |  |  |
| No | 1078/20,496 | 1 (ref.) |
| Yes | 52/590 | 1.46 (1.09-1.97) |
| **Non-mental diagnosed long-term sickness absence 1-yr prior to baseline** |  |  |
| No | 947/18,193 | 1 (ref.) |
| Yes | 183/2893 | 1.07 (0.91-1.27) |

Conditional logistic regression analyses were adjusted for the following variables at year 0: age, sex, chronic disease, psychological distress, anxiety, BMI, alcohol consumption, shift work, employment contract, occupational grade, work-unit size, work-unit temporary employment, work-unit gender distribution, as well as data-cycle no.
