# Supplementary material for: Workplace discrimination as risk factor for long-term sickness absence: Longitudinal analyses of onset and changes in workplace adversity
Source: PLoS One. 2021 Aug 5;16(8):e0255697. doi: 10.1371/journal.pone.0255697 (PMC8341535; doi:10.1371/journal.pone.0255697)
Supplement: S3 Table — Onset of workplace discrimination and odds for turnover following exposure. (DOCX) [file pone.0255697.s006.docx]

**S3 Table. Supplementary analysis. Onset of workplace discrimination and odds for turnover following exposure.**

|  | **Turnover** | |
| --- | --- | --- |
|  | Cases/No. of observations | OR (95% CI) |
| **Workplace discrimination** |  |  |
| Non-discriminated | 2579/37,440 | 1 (ref.) |
| Onset of discrimination | 193/1973 | 1.34 (1.15-1.56) |

Conditional logistic regression analyses were adjusted for the following variables at year 0: age, sex, chronic disease, psychological distress, anxiety, BMI, alcohol consumption, shift work, employment contract, occupational grade, work-unit size, work-unit temporary employment, work-unit gender distribution, as well as data-cycle no.
