# Supplementary material for: Workplace discrimination as risk factor for long-term sickness absence: Longitudinal analyses of onset and changes in workplace adversity
Source: PLoS One. 2021 Aug 5;16(8):e0255697. doi: 10.1371/journal.pone.0255697 (PMC8341535; doi:10.1371/journal.pone.0255697)
Supplement: S4 Table — Changes in workplace discrimination and odds for concurrent and 1-year lagged long-term sickness absence. (DOCX) [file pone.0255697.s007.docx]

S4 Table. Sensitivity analysis by using imputed data on missing covariates. Changes in workplace discrimination and odds for concurrent and 1-year lagged long-term sickness absence.

|  | Cause of | Concurrent  sickness absence^a^ | | 1-year lagged  sickness absence^b^ | |
| --- | --- | --- | --- | --- | --- |
|  | sickness absence | Observations | OR (95% CI) | Observations | OR (95% CI) |
| Discrimination vs.  no discrimination | Mental | 6964 | 1.61 (1.33-1.95) | 6708 | 1.01 (0.83-1.23) |
| Discrimination vs.  No discrimination | Non-mental | 27,300 | 1.07 (0.96-1.19) | 27,376 | 1.11 (0.99-1.24) |

^a^ One or more spells of absence during the exposure year. ^b^ One or more spells within one year after exposure was reported. Conditional logistic regression analyses were adjusted for age, job contract, work-unit gender distribution, work-unit size, work-unit temporary employment.
